# Large Subunit of the Human Herpes Simplex Virus Terminase as a Promising Target in Design of Anti-Herpesvirus Agents

**DOI:** 10.3390/molecules28217375

**Published:** 2023-10-31

**Authors:** Victor P. Krasnov, Valeriya L. Andronova, Alexander V. Belyavsky, Sophia S. Borisevich, George A. Galegov, Oleg F. Kandarakov, Dmitry A. Gruzdev, Olga A. Vozdvizhenskaya, Galina L. Levit

**Affiliations:** 1 Postovsky Institute of Organic Synthesis, Russian Academy of Sciences (Ural Branch), Ekaterinburg 620108, Russia; gruzdev-da@ios.uran.ru (D.A.G.); oavozdv@ios.uran.ru (O.A.V.); ca512@ios.uran.ru (G.L.L.); 2 Gamaleya National Research Center for Epidemiology and Microbiology, Ministry of Health of the Russian Federation, Moscow 123098, Russia; andronova.vl@yandex.ru (V.L.A.); g.galegov@yandex.ru (G.A.G.); 3 Engelhardt Institute of Molecular Biology, Russian Academy of Sciences, Moscow 119991, Russia; abelyavs@yahoo.com (A.V.B.); oleg.kandarakov@gmail.com (O.F.K.); 4 Ufa Institute of Chemistry, Russian Academy of Sciences, Ufa 450078, Russia; monrel@mail.ru

**Keywords:** herpes simplex virus, in vitro antiviral activity, drug resistance, high-throughput sequencing, mutation analysis, molecular docking, molecular dynamic simulations

## Abstract

Herpes simplex virus type 1 (HSV-1) is an extremely widespread pathogen characterized by recurrent infections. HSV-1 most commonly causes painful blisters or sores around the mouth or on the genitals, but it can also cause keratitis or, rarely, encephalitis. First-line and second-line antiviral drugs used to treat HSV infections, acyclovir and related compounds, as well as foscarnet and cidofovir, selectively inhibit herpesvirus DNA polymerase (DNA-pol). It has been previously found that (*S*)-4-[6-(purin-6-yl)aminohexanoyl]-7,8-difluoro-3,4-dihydro-3-methyl-2*H*-[1,4]benzoxazine (compound **1**) exhibits selective anti-herpesvirus activity against HSV-1 in cell culture, including acyclovir-resistant mutants, so we consider it as a lead compound. In this work, the selection of HSV-1 clones resistant to the lead compound was carried out. High-throughput sequencing of resistant clones and reference HSV-1/L_2_ parent strain was performed to identify the genetic determinants of the virus’s resistance to the lead compound. We identified a candidate mutation presumably associated with resistance to the virus, namely the T321I mutation in the UL15 gene encoding the large terminase subunit. Molecular modeling was used to evaluate the affinity and dynamics of the lead compound binding to the putative terminase binding site. The results obtained suggest that the lead compound, by binding to pUL15, affects the terminase complex. pUL15, which is directly involved in the processing and packaging of viral DNA, is one of the crucial components of the HSV terminase complex. The loss of its functional activity leads to disruption of the formation of mature virions, so it represents a promising drug target. The discovery of anti-herpesvirus agents that affect biotargets other than DNA polymerase will expand our possibilities of targeting HSV infections, including those resistant to baseline drugs.

## 1. Introduction

Herpes simplex virus type 1 (HSV-1), also known as human herpes virus type 1 (HHV-1), is one of the most common human pathogens. According to experts, in 2016, 66.6% of the world’s population under the age of 49 (3.752 billion people) were seropositive to HSV-1, and the infection rate of HSV type 2 (HSV-2) in the age group of 15–49 years was 13.2% (491.5 million people) [1].

After infection, the virus establishes a latent infection, persists in the host for life in the latent form, and periodically undergoes reactivation; at the same time, the frequency of recurrences, their severity, and duration depend on the immune status of the virus carrier. HSV-1 usually causes orofacial herpes [2], but can also lead to more severe diseases such as stomatitis, gingivitis, keratitis, cranial neuritis, potentially life-threatening generalized herpes infections, meningitis, and encephalitis [3].

Analysis of the global burden of genital herpes (GH) demonstrates that in 2016, 187 million people between the ages of 15 and 49 years (5.0% of the population) had experienced at least one episode of GH, of which 178 million cases (95%) were associated with HSV-2 and 9 million (5%) with HSV-1 [4]. Nevertheless, genital HSV-1 infection is becoming more common [5,6,7]. According to Japanese experts, while in men HSV-1 is the cause of GH only in 10% of cases, in women it is the cause in 45%, and the primary infection of GH in women is associated with HSV-1 in 60% of cases [8].

**Figure 1:**
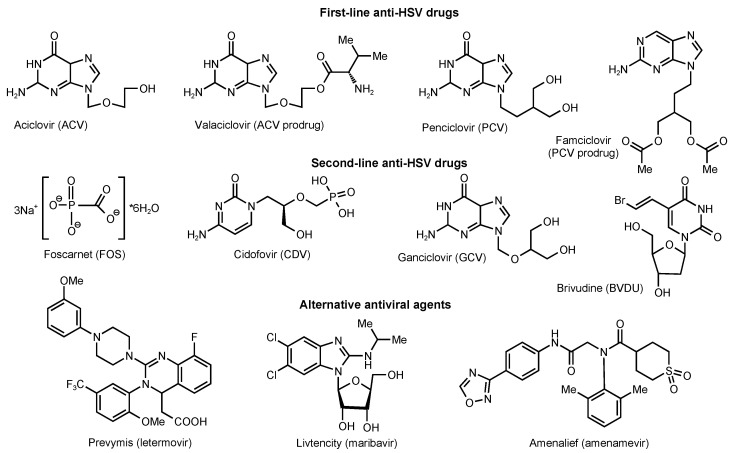
Chemical structures of approved anti-herpesvirus agents. All these anti-herpesvirus agents are inhibitors of DNA polymerase [14].

The standard practice for the prevention and treatment of HSV infections is chemotherapy based on etiotropic nucleoside-modified antiherpetic drugs such as acyclovir (ACV), its prodrug valaciclovir (L-valyl ester of ACV), and famciclovir (famvir, a prodrug of penciclovir (PCV)), which are the first-line treatment drugs (Figure 1). However, the widespread use of these drugs has led to a new problem, namely, the development of antiviral drug resistance, primarily in immunocompromised patients (HIV-infected individuals and recipients of various transplants), in whom relapses not only develop more often and are more severe but have a longer course [9,10,11,12], which requires long-term antiviral chemotherapy [13].

In order to exhibit biological activity, ACV and PCV must be converted to triphosphate in the cell. Phosphorylation of these compounds is catalyzed by the viral thymidine kinase (TK, pUL23) to the monophosphate forms [15], and by cellular kinases to di- and triphosphates [16,17]. The inclusion of ACV triphosphate in the replicating chain of viral DNA stops the synthesis by the termination mechanism, and PCV triphosphate, due to the 3′-hydroxy group, causes a slowdown in elongation and, ultimately, the cessation of viral DNA elongation. In addition, the triphosphate forms of ACV and PCV are competitive inhibitors of viral DNA polymerase (DNA-pol, pUL30) [14,16,17]. In accordance with the mechanisms of action of modified nucleosides described above, viral mutations leading to the development of antiviral drug resistance are localized either in the UL23 gene (TK, 95% of cases) or in the UL30 gene [18,19].

To suppress the reproduction of HSV strains resistant to commonly used modified nucleosides, antiviral drugs with an alternative mechanism of action are required [20]. A pyrophosphate analog, foscarnet (FOS, trisodium phosphonoformate) (see Figure 1), is currently licensed for the treatment of ACV- and PCV-resistant HSV. There is also extensive clinical experience with the use of the nucleotide analog cidofovir (CDV) for this purpose when multidrug resistance to modified nucleosides and FOS has been simultaneously developed [21]. FOS without prior phosphorylation disrupts the functioning of viral DNA-pol by inhibiting the cleavage of pyrophosphate from nucleoside triphosphate [22]. For the manifestation of antiviral activity, CDV as a derivative of cytidine nucleotide needs to be phosphorylated, but only by cellular enzymes with the formation of diphosphate, which acts as an inhibitor and alternative substrate for viral DNA-pol [23]. Since FOS and CDV are independent of HSV TK, they retain the ability to effectively inhibit the reproduction of HSV strains in which resistance to ACV is due to mutations in the TK UL23 gene. However, DNA-pol is the biotarget of all first- and second-line antiherpetic drugs; therefore, cross-resistance between these drugs may develop due to mutations in the UL30 gene [24,25,26].

It should be emphasized that FOS and CDV are used only in especially severe cases when first-line therapy is ineffective. Such limited use for the treatment of HSV infections is associated with the toxicity of second-line drugs, primarily those with a high risk of acute nephrotoxicity [27].

Considering all of the above, it can be concluded that there remains a need for alternative anti-HSV agents that have a wider therapeutic window, are able to more effectively suppress virus shedding and prevent recurrence of infections, and are also capable of suppressing resistant HSV strains [28].

In the last 10 years, the following drugs have entered clinical practice (see Figure 1): Prevymis™ (2017, Merck Sharp & Dohme B.V., Haarlem, The Netherlands) based on letermovir, an inhibitor of human cytomegalovirus (HCMV) terminase for the prophylaxis of cytomegalovirus infections in adult HCMV-seropositive recipients (R+) of an allogeneic hematopoietic stem cell transplant (effective only against HCMV, but not any other herpes viruses); orphan drug Livtencity^®^ (2021, Takeda, Tokio, Japan) based on maribavir, an inhibitor of HCMV protein kinase pUL97 [29,30] to suppress HCMV infection with multidrug resistance (in vitro activity against the Epstein-Barr virus (EBV) has also been shown); and Amenalief^®^ (Maruho, Osaka, Japan) based on amenamevir, a helicase-primase complex inhibitor of HSV and varicella zoster virus (VZV), licensed only in Japan in 2017 for the treatment of herpes zoster, and from 24 February 2023, for the treatment of recurrent herpes simplex virus [31,32].

**Figure 2:**
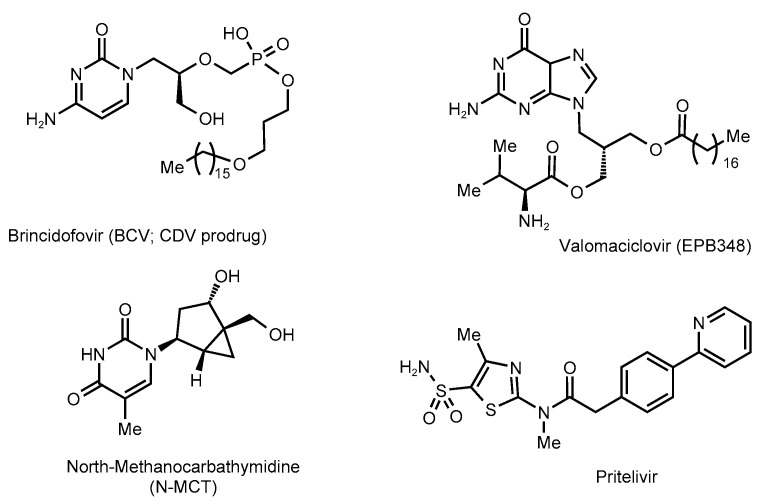
Chemical structures of antiviral agents in clinical trials.

Currently, several more drugs are undergoing clinical trials for safety and efficacy against herpesvirus infections (Figure 2).

Brincidofovir (BCV; Chimerix, Durham, NC, USA) is an oral lipid-conjugated prodrug of CDV. BCV has the same spectrum of activity as CDV and is active against double-stranded DNA (dsDNA) viruses, including all herpesviruses [33,34,35], adenoviruses [36], orthopoxviruses [37], and polyomaviruses [38].

Valomaciclovir (EPB-348) was initially developed by Epiphany Biosciences (San Francisco, CA, USA) and since 2006 developed jointly with Medivir (Huddinge, Sweden). This prodrug form of omaciclovir ((*R*)-9[4-hydroxy-2-(hydroxymethyl)butyl]guanine) is active in vitro against HSV-1, HSV-2, VZV, EBV, and HHV-6, but not active against HCMV and HHV-7, as well as HHV-8 (KSHV) [39]. A Phase 2b Trial (ClinicalTrials.gov Identifier: NCT00831103) was conducted to evaluate the safety and efficacy of EPB-348 among adult immunocompetent patients with an acute episode of herpes zoster [40]. It has been found that EPB-348 is well tolerated by adults and is not inferior to valaciclovir [41]. However, it needs to be phosphorylated by viral TK, and in the form of triphosphate it is a competitive inhibitor of viral DNA-pol, so mutations associated with VZV resistance to EPB-348 are localized in the UL23 TK gene and also confer cross-resistance to ACV [42].

North-Methanocarbathymidine (N-MCT; N&N Scientific Co., Potomac, MD, USA) is active against HSV-1, HSV-2, EBV, and HHV-8 (but not HCMV), including ACV-resistant HSV-1, and also has potent antiviral activity against both cowpox and vaccinia viruses, including CDV-resistant vaccinia virus [43,44]. It is an analog of thymidine with a *pseudo*-sugar moiety. In the cell, it is sequentially phosphorylated by viral TK to mono- and diphosphate forms, and then by cellular enzymes to 5′-triphosphate, which inhibits viral DNA synthesis [44,45]. In 2016, clinical trials of N-MCT for evaluating the safety and plasma levels of N-MCT in normal patients (ClinicalTrials.gov Identifier: NCT02778386) were announced [46], and the status of an orphan drug for the treatment of neonatal herpes infection was designated to N-MCT [47].

Pritelivir (BAY 57-1293, AIC316; AiCuris, Wuppertal, Germany), similarly to amenamevir, is a selective inhibitor of the HSV helicase-primase enzymatic complex [48]. Unlike currently used nucleoside analogs, pritelivir does not require activation by viral kinases; therefore, mutations that confer ACV resistance do not affect the efficacy of pritelivir both in vitro [49] and in vivo [50]. In addition, since DNA-pol is not a biotarget of pritelivir, FOS-resistant HSV strains also remain susceptible to pritelivir [49]. Currently, a phase 3 trial on the efficacy and safety of pritelivir tablets for treatment of ACV-resistant mucocutaneous HSV infections in immunocompromised subjects (PRIOH-1) versus foscarnet is currently underway (ClinicalTrials.gov Identifier: NCT03073967) [51], as well as the electrocardiographic effects of pritelivir in healthy subjects (ClinicalTrials.gov Identifier: NCT05671029) [52].

Thus, the search for new original compounds, the activity of which against the herpes virus is due to the effect on targets other than DNA-pol, remains relevant and is currently being actively carried out by leading pharmaceutical companies.

**Figure 3:**
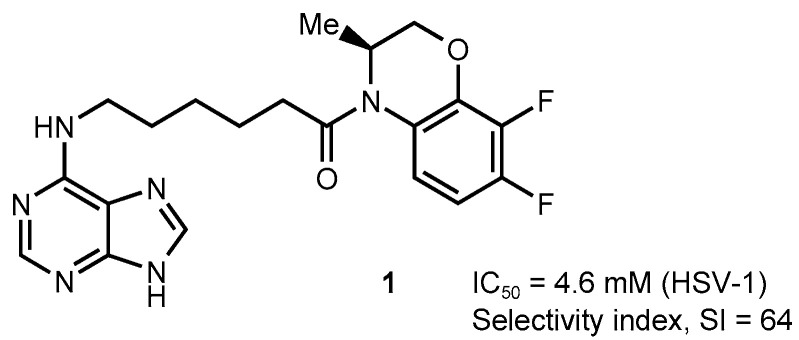
Structure of compound **1**.

For a number of years, we have been conducting research on the synthesis of purine conjugates, which are potential antiviral and antibacterial agents [53,54,55,56,57]. It has been found that (*S*)-4-[6-(purin-6-yl)aminohexanoyl]-7,8-difluoro-3,4-dihydro-3-methyl-2*H*-[1,4]benzoxazine] (compound **1**, Figure 3) is a selective inhibitor of HSV-1, including an ACV-resistant HSV-1 strain [53], so this conjugate was chosen as the lead compound.

As we reported earlier [58], using the time-of-addition assay, we determined the time period for which it is possible to delay the addition of lead compound **1** to the experimental system without losing its antiviral activity. According to the results obtained, the crucial time of compound **1** addition coincided with that of ACV and FOS, reference drugs with a well-known mechanism of action (both compounds are HSV-1 DNA-pol inhibitors). Thus, it can be assumed that compound **1** interferes with the life cycle of HSV-1 during viral DNA synthesis. It is important to note that modifications of the structure of compound **1** and a number of related compounds (e.g., the introduction of a sugar or *pseudo*-sugar residue into the molecule) led to a significant decrease in antiviral activity. Moreover, the equally high activity of compound **1** and its structural analogs against HSV-1 and ACV-resistant HSV-1 strain [53] suggested that these compounds do not need to be activated by viral TK, and the mechanism of their action is different from that of ACV. However, further studies are needed to identify a possible biotarget of compound **1**. 

For this purpose, in this study, we obtained an HSV-1 population with reduced sensitivity to compound **1** and managed to isolate three resistant clones from it. To identify mutations leading to resistance to compound **1**, high-throughput sequencing of their genomes was carried out, candidate mutations were identified, and the affinity of compound **1** to putative binding sites was assessed using molecular modeling.

## 2. Results

### 2.1. Preparation of Compound ***1***-Resistant HSV-1 Population

Delaying the emergence of drug resistance in the virus potentially increases the effectiveness of chemotherapy and improves the clinical outcomes of recurrent herpes infection. Therefore, the rate of formation of a population resistant to an antiviral agent is one of its important characteristics, and the identification of mutations leading to drug resistance makes it possible to determine the biotarget of an antiviral agent and the mechanism of its action. The criterion used to define a virus population as resistant was an IC_50_ value ten-fold greater than that for the parental wild-type HSV-1/L_2_ tested in the same assay [59].

To obtain a compound **1**-resistant population, a series of successive passages of the HSV-1/L_2_ reference strain in the presence of compound **1** was carried out. Only after 5 successive passages in the presence of compound **1** at a concentration of 4 µg/mL, the sensitivity of the virus decreased by 1.5 times, which made it possible to increase the concentration of the agent in the support medium to 6 µg/mL during passage 6. The sensitivity of the material of passage 6 decreased by a factor of two, and passage 7 was carried out in the presence of compound **1** at a concentration of 8 µg/mL (~4 IC_50_). As a result, the sensitivity of the obtained virus-containing material decreased to 7.8 µg/mL (4 IC_50_). Passage 8 was carried out in the presence of 10 µg/mL of compound **1** in the support medium, and in the next passage, the concentration was increased to 12 µg/mL.

The sensitivity to compound **1** of the population (HSV-1/L_2_/R**_1_**) obtained after nine successive passages was five-fold lower than the sensitivity of the original population (IC_50_ 9.75 and 1.95 µg/mL, respectively). It is important to note that the ACV-resistant population formed much faster when passaged under the same conditions. As we have shown previously, during serial passages of HSV-1/L_2_ under similar conditions, in order to form an ACV-resistant population with an IC_50_ value 32 times higher than that of the initial population, it was sufficient to carry out four passages to increase the ACV concentration from 2.5 µg/mL (6 IC_50_ in passage 1) to 10 µg/mL (25 IC_50_ in passage 4) [60].

The resulting HSV-1/L_2_/R**_1_** population with five-fold reduced sensitivity to compound **1** was used to isolate compound **1**-resistant virus clones. Cloning was performed as described in the Section 3. After that, the inhibitor sensitivity of clones selected from the population was determined. Three of them with an IC_50_ of 20 µg/mL, i.e., 10 times the IC_50_ of the original population, were selected for further studies.

To test whether the resulting compound **1**-resistant HSV-1 clones and the population as a whole are also resistant to the available basic and well-known antiherpetic drugs (ACV and related compounds), as well as BVDU, AraA, CDV, and FOS, in vitro, we performed a comparative analysis of their sensitivity and that of the parent HSV-1/L_2_ population to these antiviral agents. Table 1 shows the results of studying the sensitivity (in terms of IC_50_ values) of the HSV-1/L_2_ reference strain, the HSV-1/L_2_/R**_1_** population, and three compound **1**-resistant HSV-1 clones to compound **1** and approved antiherpetic drugs in the Vero E6 cell culture. As can be seen from the data in Table 1, HSV-1/L_2_/R**_1_** and three clones retained sensitivity both to TK-dependent inhibitors of HSV reproduction (ACV, PCV, GCV, BVDU, and IDU) and to compounds that do not require prior activation by viral TK (FOS and Ara-A). The IC_50_ values were equal or insignificantly (by a factor of 1.5–2) differed from those of the parental strain HSV-1/L_2_. Since none of the clones resistant to compound **1** were cross-resistant to ACV, other modified nucleosides, CDV, or FOS, these results indirectly indicate that the mechanism of action of compound **1** on HSV-1 reproduction is different from the mechanism of action of all of the above DNA-pol inhibitors. The results of these studies may also serve as a basis for the development of selective inhibitors of the HSV-1 terminase complex in order to expand the ability to target HSV infections that are resistant to basic antiherpetic drugs.

### 2.2. High-Throughput Sequencing of Resistant Clones

To identify mutations in the herpesvirus genome resulting in the acquisition of resistance to compound **1**, Vero E6 cells were infected by the initial virus strain (HSV-1/L_2_) sensitive to the compound **1** (referred to hereafter as wild type) and three resistant virus clones. Total DNA was isolated and sequenced by high-throughput sequencing. The total number of reads varied between 5.11 × 10^6^ and 6.41 × 10^6^ for different DNA samples. Bioinformatics analysis using two programs [61] demonstrated that between 15% and 24% of reads were mapped onto reference herpesvirus genome (GenBank accession No. BK012101). The total number of reads and percentage of the mapping are represented in Table 2.

To identify candidate mutations that could be responsible for acquisition of compound 1-resistant phenotype, a comparison of reads mapping on reference genome in four DNA samples was performed. To narrow the list of candidate mutations, the following criteria were used: (a) mutation must be present in all three resistant strains but not in wild-type virus; (b) mutation must be present in protein-coding region; (c) the mutation must result in an amino acid change; silent mutations were not considered.

The search for mutations according to these criteria revealed the presence of mutations in five proteins that potentially could result in resistance to compound **1**. These mutations are listed in Table 3.

A comparative analysis of the genome of the HSV-1/L_2_ reference strain and the genomes of three HSV-1 clones isolated from a population that had undergone a series of passages in the presence of compound **1** and were characterized as resistant, revealed five mutations leading to amino acid substitutions in all three clones.

I. The UL2 gene encodes uracil-DNA glycosylase [62], whose function is to eliminate lesions in DNA, such as spontaneous deamination of cytosine or the misincorporation of dUMP during replication [63]. According to the literature data, pUL2 is not required for virus growth in cell culture: deletion of this gene does not impair virus replication in vitro [62,64,65]. Therefore, it is extremely unlikely that any mutation in this protein, including the detected R19H substitution, could be important for the development of drug resistance, regardless of its effect on the phenotypic properties of pUL2.

II. The UL30 gene product is a catalytic subunit of DNA-pol of HSV, the loss of functional activity of which is lethal to the virus. It should be noted that the identified E799D substitution is localized in the non-conserved region VI–III of pUL30 (fingers domain). However, the literature describes mutations P797T, E798K, and L802F located in the same region [66] and associated with resistance to modified nucleosides and pyrophosphate analogs of phosphonoacetic acid and FOS. In contrast to the above amino acid substitutions, glutamic and aspartic acids are structurally very close (they are homologs) and are characterized by similar properties. In addition, the sensitivity of all three clones to inhibitors of DNA-pol, namely, ACV and related compounds (GCV, PCV, BVDU, and IDU), as well as CDV, Ara-A, and FOS, was not changed (Table 1). Taken together, these data indicate a low probability of an association between the E799D substitution and a decrease in the sensitivity of the virus to compound **1**.

III. The UL37 gene encodes a multifunctional tegument component, the phosphoprotein pUL37, which forms a complex with another tegument protein pUL36, which plays a key role in the early stages of the viral maturation pathway that leads to the emergence of an infectious virion. pUL37 is absolutely essential for HSV reproduction. A virus with a null mutation in the UL37 gene was unable to replicate in Vero E6 cells. The production of DNA-containing capsids was not disturbed, therefore, pUL37 is not required for DNA cutting or packaging. However, the release of nucleocapsids from the nucleus slowed down, and nucleocapsids, devoid of a lipid envelope, which they should have received when incorporated into transport vesicles originating from the *trans*-Golgi network (TGN) [67], accumulated in the cytoplasm, and their subsequent maturation into virions was not implemented [68].

The C-terminus of pUL37 (amino acid residues (AARs) 568–1123) is responsible for binding to pUL36 (VP1/2) [69,70], thus providing pUL36-mediated binding of pUL37 to the capsid [71]. A mutation identified in the UL37 gene results in the replacement of R790H at the C-terminus of pUL37. Amino acid R790 is part of a domain (AARs 578–899) that interacts with dystonin/BPAG1 (spectrplakin protein), which is known as a cytoskeletal linker involved in nucleocapsid stabilization and its transport along microtubules in the cytoplasm during release from infected cells [72]. However, as noted above, compound **1** does not affect the late stages of HSV reproduction.

The amino acids arginine and histidine are not structurally related but contain nitrogen-containing positively charged side chains, so it could be assumed that such a substitution may be associated with the development of drug resistance in the virus. However, HSV-2, in contrast to HSV-1, contains histidine at position 790 and, despite this, is sensitive to compound **1**.

IV. The US7 gene encodes glycoprotein I (gI), which forms a heterodimeric complex with glycoprotein gE on the surface of the virion. It is not involved in the processes of adsorption and penetration of the virus into the cell [73] and the release of the virus from the cell into the extracellular space, but it is an important mediator of the spread of the virus to neighboring uninfected cells through lateral intercellular junctions (the so-called “cell-to-cell spread”, CCS), the result of which is the formation of syncytium. Directed traffic of intracellular viral particles to lateral junctions is provided by gE/gI [74]. The propensity of HSV to direct CCS appears to be highly dependent on cell type. In polarized cells (epithelial cells and neurons), in contrast to non-polarized ones, CCS is predominant; therefore, gI is important for establishing latent infection in the trigeminal ganglia during primary infection and for anterograde transmission of HSV from neuronal to epithelial cells during relapse [75,76,77,78].

Although comparative analysis of the sequences of HSV-1 strains KOS, F, and 17 (GenBank: JQ780693.1), and 23 clinical isolates (GenBank accession nos. from AJ626527 to AJ626556) showed that at position 165 in the US7 gene, as in strain L_2_, as well as in HSV-2 (strain HG52, NCBI Reference Sequence: NC_001798.2), alanine was mapped, it cannot be ruled out that the substitution of nonpolar hydrophobic alanine for polar hydrophilic threonine at position 165 found in gI can hinder or even exclude passaging the virus from cell to cell. However, it is unlikely that the A165T mutation in the US7 gene is associated with the development of virus resistance to compound **1** for the following reasons. The results we obtained earlier allowed us to conclude that compound **1** does not affect the later stages of the virus life cycle [58], and, accordingly, its mechanism of action is most likely not related to gI. This assumption is also confirmed by the data, according to which the reproduction of gI-negative mutant viruses in human fibroblasts is not suppressed, and the rate of penetration of mutant viruses and wild-type viruses into fibroblasts is the same. Although the rate of spread of such mutant viruses from cell to cell was significantly slower than that of wild-type HSV-1, even the deletion of the US7 gene is not crucial for the survival of the virus [79]. In addition, the release of HSV in the culture of non-polarized Vero cells (African green monkey kidney fibroblasts), which we used in the study of antiviral activity, occurs mainly through the apical rather than lateral cell surfaces. Both gE and gI mutant viruses are reported to produce smaller plaques in Vero cells than the wild-type virus. However, this effect was less pronounced than in human fibroblasts, and the number of plaques produced by mutant viruses and wild-type viruses did not differ. So, CCS of wild-type HSV-1 is more efficient than the propagation of gE- or gI-mutant viruses in fibroblasts [80], but mutant viruses have not lost the ability to reproduce. Therefore, it is difficult to assume that even complete inhibition of the gI function can provide a significant suppression of its reproduction in this cell culture.

Thus, the most likely candidate for the role of a mutation that causes resistance to compound **1** is a mutation in the UL15 gene.

V. The UL15 gene encodes a large subunit of the terminase complex, whose functions include recognition, the translocation of concatemeric dsDNA of HSV into a preformed procapsid through the dodecameric portal, cleaving it at the exact location to release unit-length genomes bound head-to-tail, and providing energy for the processes of packaging and cutting dsDNA. These processes are one of the key stages of the reproductive cycle of HSV [81,82] and their implementation requires at least seven virus-encoded proteins: pUL15, pUL28 (small terminase subunit), pUL33, structural proteins that make up the capsid, namely, pUL6 (portal protein), pUL17, and pUL25 (ensures the correct orientation of the procapsid relative to the terminase complex [83], as well as pUL32 (probably as a chaperone-like protein) which modulates the disulfide bond formation during the procapsid assembly and maturation [81,84].

**Figure 4:**
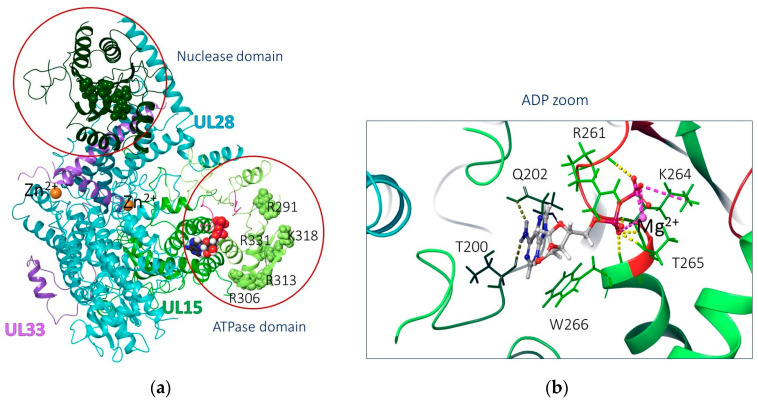
(**a**) UL15–UL28–UL33 heterotrimer architecture: green color corresponds to UL15 subunit; blue and purple, UL28 and UL33, respectively. Zinc cations are shown as orange balls. Significant regions of the protein are marked with red circles. (**b**) ADP binding in the ATPase domain: hydrogen and salt bridges are shown in yellow and purple, respectively. Magnesium cation is shown in pink. Secondary structures of Walker A (AARs 258–265) and Walker B (AARs 352–357) motifs are shown in red.

The HSV dsDNA packaging biomotor, which performs the function of cleavage and encapsidation of viral DNA using ATP, is a hexameric ring, each subunit of which consists of the main multifunctional motor protein pUL15 (large subunit, the ATPase/nuclease) and two regulator/fixer proteins, namely pUL28 (small subunit) and pUL33 (Figure 4) [82].

It is pUL15 that includes two domains: the C-terminal nuclease domain (AARs 479–735), which cuts viral DNA, and the N-terminal ATPase domain (AARs 253–413), which hydrolyzes ATP, providing energy for cutting and transferring the genome [85], and also forms the surface of the inner channel. The concatemeric DNA enters the central inner channel of the hexameric motor ring formed by the N-terminal domains of six pUL15s. Its translocation is initiated upon binding and hydrolysis of ATP by *trans*-acting arginine fingers, accompanied by significant conformational transition of the ATPase [82]. After binding the first ATP, R346 is inserted into the ATP-binding pocket of the neighboring subunit and interacts with γ-phosphate [86].

The T321I amino acid substitution that we have determined is located in the N-terminus of pUL15 and is not part of the conservative Walker A (AARs 58–65) and B (AARs 352–357) motifs and the conservative HSV-1 nuclear localization signaling motif pUL15 (NLS) (AARs 183–189) [87,88], but is located in close proximity to the functional AARs R291, K292, R306, R313, K318, and R331 that form the surface of the central channel of the terminase complex [82], which binds and moves negatively charged DNA into a limited region procapsid space through the dodecameric portal [89].

It could be assumed that the replacement of threonine containing a polar uncharged hydrophilic side chain with isoleucine with a nonpolar hydrophobic side chain could affect the spatial structure of the region of the pUL15 N-terminus without significantly disturbing the functional activity of the ATPase catalytic center, leading to the development of virus resistance to compound **1**. To determine the potential binding site of compound **1**, a molecular docking procedure was carried out.

### 2.3. Molecular Modeling Study

#### 2.3.1. Biological Target Analysis

An analysis of the results of biological experiments allows us to consider terminase as a potential biological target. As mentioned above, the herpesvirus terminase is the central component of the viral genome packaging and is the main target for the development of antiviral drugs.

The UL15 subunit contains 735 AARs and is more than 50% conserved in the *Herpesviridae* family [82]. It contains 5 functional domains, including the ATPase domain (AARs 253–413) and the nuclease domain (AARs 479–735). The ATPase domain of pUL15 converts the chemical energy obtained as a result of the hydrolysis of the ATP γ-phosphate bond into mechanical force. The process involves a series of conformational changes in the motor building block. It is believed that functional AARs R291, K292, R306, R313, K318, and R331 are located in the central channel of the DNA-binding domain [82]. The ATPase active site includes Walker A and Walker B motifs (or P-loops). Walker A motif coordinates ATP binding and hydrolysis [87]. The presence of Mg^2+^ cation was also mentioned [87]; on the one hand, it is coordinated with AARs, and on the other hand, with ATP (Figure 4b).

#### 2.3.2. Molecular Docking Results

The molecular docking procedure for compound **1** was carried out in various protein cavities and potential binding sites. The ATP domain (ATP-D), the central channel (CC), the movable loop located in close proximity to the central channel, and the Walker B motif were considered as binding sites.

##### Docking to the ATP Domain

**Figure 5:**
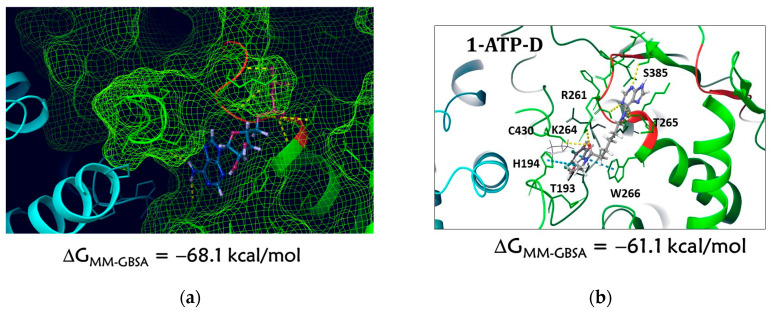
Visualization of the location of ADP and compound **1** molecules in the ATP domain: (**a**) ADP location corresponds to PDB ID: 6M5V [82]; (**b**) docking position of compound **1** at the binding site, **1**–ATP-D ligand–protein complex. Green color of secondary structure corresponds to UL15 subunit, blue color to UL28 subunit. The dashed green lines show the surface of the UL15 subunit.

An analysis of the results of molecular docking suggests that the studied ligand (compound **1**) can bind in the ATP domain. Compound **1** is located in the ATP domain close to the Walker loops, forming intermolecular interactions with a number of AARs: the purine fragment of the ligand lies in the polar cavity near the Walker A motif with the formation of hydrogen bridges with the atoms of amino acids S385, T265, and R261, and the fluorine-containing aromatic fragment separated by a long linker is located in a hydrophobic cavity and forms a π-π stacking interaction with W266 and H194 (Figure 5). In addition, hydrogen bridges are registered between the atoms of the ligand and the AARs C430 and R264.

Additional energy parameters characterizing the affinity of compound **1** for the binding site are presented in the Supplementary Materials (see Table S1).

##### Docking to Alternative Binding Sites

**Figure 6:**
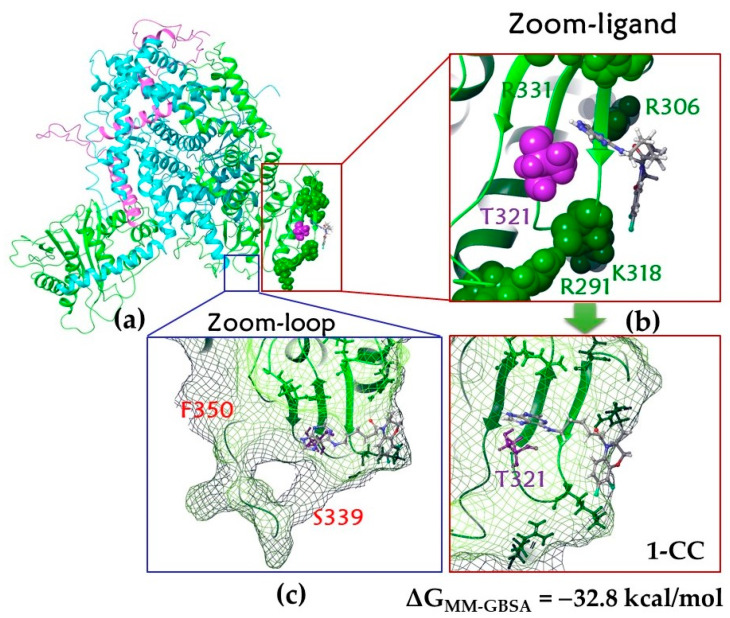
UL15–UL28–UL33 complex: (**a**) visualization of the secondary structure of the complex; (**b**) the result of the ligand affinity assessment for the central channel (key AARs involved in DNA promotion are shown in green; T321 is shown in purple); (**c**) loops 339–350, inside which the ligand could theoretically fit. Green color of the secondary structure corresponds to UL15 subunit; blue and purple, to UL28 and UL33, respectively.

Amino acid T321, along with AARs R291, K292, R306, R313, K318, and R331, is located in the central channel involved in DNA promotion (Figure 6a). Theoretically, a molecule could be located in such a small recess. However, the results of molecular docking indicate a low affinity of compound **1** for the putative site (Figure 6b). The binding energy is −32.8 kcal/mol, which is almost two times higher than the binding energy of the ligand in the ATP binding site.

Additional analysis of the surface of the protein complex allows us to consider the likely site of ligand binding located under or near the loop including AARs from 339 to 350, which is in close proximity to AARs 352–357 of the Walker B motif (Figure 6c). Taking into account the descriptor features of the ligand, it can be assumed that compound **1** with the optimal linker length can be located in the cavity under the indicated loop. Then, we carried out the procedure of molecular docking into the space of a protein limited by AARs, among which is the mutating amino acid T321.

**Figure 7:**
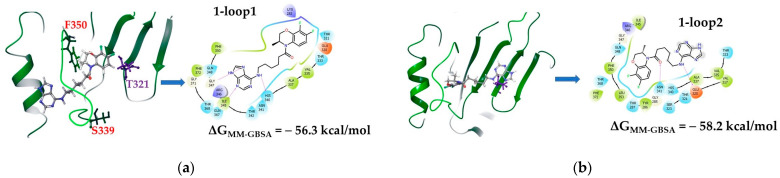
Results of molecular docking of compound **1** into the protein space near loops 339–350: (**a**) location of the ligand under the loop in which the fluorine-containing fragment is located next to T321; (**b**) alternative location of the ligand, the purine fragment is closer to T321.

The visualization of the calculations showed that the studied ligand, compound **1**, can bind in this cavity with the formation of a number of intermolecular interactions, including a hydrogen bridge directly with T321. Figure 7 shows two positions for compound **1**. The positions differ in the location of the purine fragment: in the first case, the molecule lies “under the loop” (Figure 7a), and the purine fragment is located in the vicinity of the α-helix (AARs 365–373). Hydrogen bridges are registered between the hydrogen atoms of the purine fragment and G347, Q367, and H340 (Figure 7b). The 7,8-difluorobenzoxazine fragment is located at a distance of about 3 Å from T321. However, such an arrangement of the ligand leads to a number of undesirable clash interactions, including those with T321 (see the Supplementary Materials, Table S1). In another position, the aromatic fragments lie in opposite directions: the fluorine-containing fragment is closer to the α-helix, and the purine fragment is closer to T321 (Figure 7b). There are no clash interactions in this position (Table S1). Hydrogen bridges are registered between the carbonyl oxygen of the ligand and N341, and between the amino nitrogen of the linker and Q320 (Figure 7).

In both positions, the molecule of compound **1** is quite close to T321. Probably, the arrangement of the ligand described above can affect the secondary structure of the protein, especially the mobile loops.

Nevertheless, analysis of the results of the molecular docking procedure does not allow one to reliably determine the binding site of compound **1**. We can only note the low probability of ligand binding in the central channel, on the one hand, and the comparable affinity of the ligand for the ATP domain and the alternative binding site, on the other. To assess the behavior of compound **1** in the considered binding sites, as well as the degree of influence on the secondary structure of the complex, we performed a series of molecular dynamics simulations. The geometric parameters of the ligand–protein complexes obtained as a result of molecular docking procedures were used as starting models.

#### 2.3.3. Molecular Dynamic Results

Four ligand–protein complexes were subjected to the molecular dynamics procedure: **1**–ATP-D corresponds to the location of the ligand in the ATP domain; **1**–SS, in the central channel; and **1**–loop1 and **1**–loop2, two docking positions of the ligand next to loops 339–350.

##### Molecular Dynamics of the **1**–ATP-D Complex

An analysis of the root-mean-square deviation (RMSD) of atomic positions (see the Supplementary Materials, Figure S1A) shows that the system has equalized by the end of the simulation. Fluctuations for protein are acceptable in the range from 1 to 3 Å. For a ligand, the amplitude may be deeper, but noticeable fluctuations indicate that the location of the ligand is constantly changing throughout the simulation. This can be seen in the oscillations in the first ns of the simulation.

An analysis of fluctuation (ligand effect) on AARs of the binding site (Supplementary Materials, Figure S2A) shows that compound **1** has a noticeable effect on UL15 subunit AARs (AARs 170–205 and 206–266) as well as on UL28 subunit AARs (AARs 701–704). This result indicates a significant shift of the ligand relative to its starting position. The graph correlates with the Protein–Ligand Contacts histogram (Supplementary Materials, Figure S3A), which shows the duration of intermolecular contacts during the entire simulation time. In this case, prolonged contacts (more than 50% of the simulation time) are recorded with AARs 700–704.

**Figure 8:**
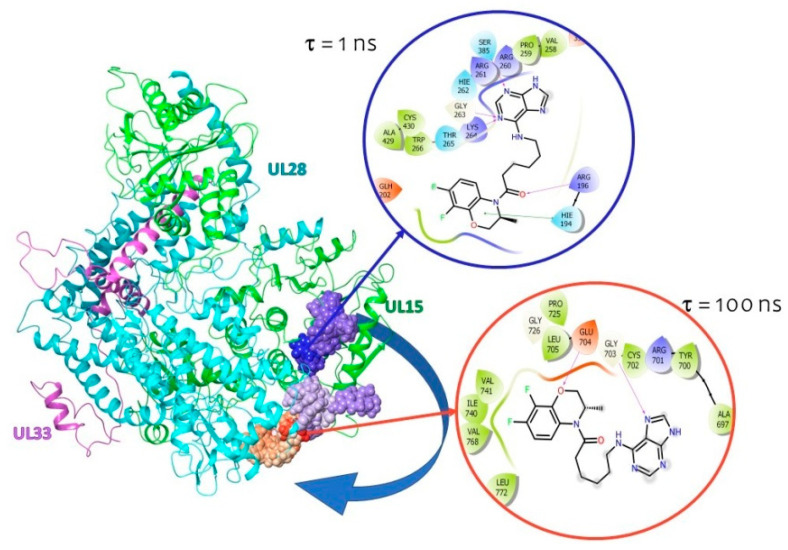
Ligand migration (at start position, ligand in blue circle) during 100 ns of simulation (at last position, ligand in red circle). The blue arrow shows the trajectory of the ligand.

Visualization (Figure 8) shows that the ligand resides at the ATP binding site for a short time and then migrates to another UL28 subunit. The location of the ligand in this part of the complex is characterized by the formation of hydrogen bonds with E704 and G703. Both amino acids belong to the UL28 subunit.

Thus, the ligand does not diffuse into the solvent, but its position relative to the protein surface changes. Then, we can assume that despite the presence of affinity for the ATP binding site, the ligand will not be retained there.

##### Molecular Dynamics of the **1**–CC Complex

An analysis of RMSD of atoms in the **1**–CC complex (see the Supplementary Materials, Figure S1B) shows noticeable ligand oscillations up to 38 ns of simulation. Further, the position of the ligand is aligned. However, as in the case of the **1**–ATP-D complex, the ligand forms multiple contacts with the AARs of the UL15, UL28, and even UL33 subunits. This also indicates (Figure S2B) that the ligand is free to move on the surface of the protein. At the same time, the position of the compound **1** molecule also shifts to the region of the UL28 subunit, forming the longest hydrogen bridges with R722 and S743 (Figure S3B). In other words, ligand binding in the central channel is unlikely.

##### Molecular Dynamics of **1**–loop1 and **1**–loop2 Complexes

The position of the ligand in both complexes is equalized only at the end of the molecular dynamic simulations (see the Supplementary Materials, Figure S1C,D). However, analysis of RMSF (Figure S2C,D) indicates that during the entire simulation time, the ligand is located in a given region of the protein, contacting AARs 283–375 of the UL15 subunit. The position of the ligand changes at the binding site, but the molecule does not leave the site itself. In the **1**–loop2 complex, the ligand molecule unfolds into a position corresponding to the **1**–loop1 complex: the purine moiety is surrounded by polar AARs Q348, H340, N341, and T342 (Figure 7a). The hydrogen bridge between the nitrogen atom of the purine fragment and G347 (Figure S3C) in the **1**–loop1 complex is maintained for more than 80% of the simulation time; in the **1**–loop2 complex, more than 50%. Hydrophobic contacts are formed between the fluorine-containing fragment of the compound **1** molecule and the hydrophobic AARs V335 and A337 (Figure S3C,D). In general, fewer intermolecular contacts were registered (Figure S3C,D) compared to the analysis of the dynamics of the **1**–ATP-D and **1**–CC complexes.

**Figure 9:**
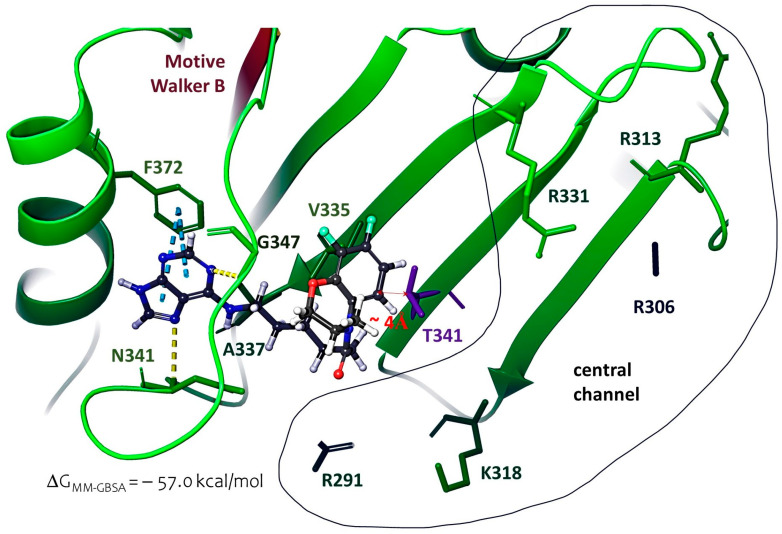
The location of the ligand at the alternative binding site of the UL15 subunit: hydrogen bridges and π-π stacking interactions are shown as yellow and blue dashed lines. The secondary structure of the Walker B motif is shown in red.

The frame clustering procedure of molecular dynamic simulations made it possible to obtain the optimal position of the ligand in the considered binding site (Figure 9). The purine fragment is located near the α-helix (AARs 365–373) and forms a π-π stacking with F372. The nitrogen atoms of the purine fragment form hydrogen bridges with N341 and G347. The hydrophobic linker is located near the hydrophobic AAR A337, a fluorine-containing aromatic fragment near V335. The mutating amino acid T341 is located at a distance of no more than 4 Å from the ligand. The binding energy of the ligand and protein is −57.0 kcal/mol.

Using the procedures of molecular docking and molecular dynamic simulations, a possible site of compound **1** binding to pUL15 was determined, namely the area under loops 339–350 near the central channel in close proximity to the mutated AAR T321.

The ligand atoms interact with AARs R291, R331, and K318, which form the surface of the central channel, and additionally with T321 (see the Supplementary Materials, Figures S2C,D and S3C,D), which means that compound **1** can affect the conformation of the side chains of these AARs. Possibly, conformational changes of the central channel AARs may block the process of translocation and/or dsDNA binding.

Biomotors are classified into three categories: linear, rotational, and revolving motors. It is known that the herpesvirus hexameric motor (terminase complex) belongs to the category of revolving biomotors. The rotational model requires that the diameter of the motor channel does not exceed 2 nm (dsDNA diameter). If the motor channel is larger, rotation will not occur because the inner surface of the channel and dsDNA will lose contact. The channel of the HSV terminase complex, 3.9 nm in diameter, is about 50% larger than the dsDNA diameter [82,90]. The large diameter of the channel allows dsDNA to revolve. It is this feature, as well as the fact that the channel is formed by the pUL15 ATPase domain, that indicates that the genome-packaging complex of HSV is a revolving biomotor [91]. Revolving motors form a special structure to drive dsDNA translation through the mechanism of a one-way traffic of genomes into procapsids without rotation. According to this model, dsDNA translocation is carried out as a result of successive actions of the subunits of the complex, and it is likely that inhibition of any of them can lead to deactivation of the hexameric terminase complex and, accordingly, its biological activity [92]. However, the question of the number of subunits that must bind to the antiviral agent (compound **1**) to block the function of the complex as a whole remains open. The answer to this question may also help to understand why HSV resistance to compound **1** is formed much more slowly than to ACV. Probably, if one subunit containing a resistance-associated mutation is included in the hexameric terminase complex, this will not be enough for the functioning of the complex as a whole. However, it is not clear how many proteins containing such a mutation in the complex are necessary to restore its activity in the presence of an inhibitor. Further research is needed to answer this question.

Viruses are obligate parasites that replicate in host cells, which greatly complicates the search for compounds that can efficiently inhibit virus replication without significantly affecting the host cell and the macroorganism as a whole. Search for new potential biotargets is one of the ways to design new antiviral agents. Our results could potentially pave the way for a targeted search for new anti-herpesvirus compounds with higher binding affinity to the multisubunit terminase complex. This complex may serve as an ideal target for the development of new anti-herpesvirus agents. It is important to emphasize that the DNA-cutting process is absent in eukaryotic cells; therefore, pUL15, which is more than 50% conserved in the *Herpesviridae* family [82], is a promising biotarget for the development of safe and efficient anti-herpesvirus drugs.

## 3. Materials and Methods

### 3.1. Compounds

Compound **1** ((3*S*)-7,8-difluoro-3,4-dihydro-3-methyl-4-[6-(purin-6-ylamino)hexanoyl]-2*H*-[1,4]benzoxazine, C_20_H_22_F_2_N_6_O_2_, M 416.42) was synthesized as described earlier [53]. The following antiviral agents were used (all from Sigma-Aldrich, St. Louis, MO, USA): acyclovir (ACV, 9-[(2-hydroxyethoxy)methyl]guanine, C_8_H_11_N_5_O_3_, M 225.20), penciclovir (PCV, 9-[4-hydroxy-3-(hydroxymethyl)butyl]guanine, C_10_H_15_N_5_O_3_, M 253.26), ganciclovir (GCV, 9-[(1,3-dihydroxy-2-propoxy)methyl]guanine, C_9_H_13_N_5_O_4_, M 255.23), (*E*)-5-(2-bromovinyl)-2′-deoxyuridine (BVDU, C_11_H_13_BrN_2_O_5_, M 333.14), idoxuridine (IDU, 5-iodo-2′-deoxyuridine, C_9_H_11_IN_2_O_5_, M 354.10), cidofovir (CDV, C_8_H_14_N_3_O_6_P, M 279.19), adenine arabinoside (Ara-A, 9-β-D-arabinofuranosyladenine, C_10_H_13_N_5_O_4_, M 267.24), and foscarnet (FOS, foscarnet sodium, CNa_3_O_5_P × 6H_2_O, M 300.04).

### 3.2. Viruses

Herpes simplex virus type 1 strain L_2_ (HSV-1/L_2_) was obtained from the State Collection of Viruses (Ivanovsky Institute of Virology, Gamaleya National Research Center of Epidemiology and Microbiology, Ministry of Health of the Russian Federation, Moscow, Russia). The virus was maintained by passaging using a growth medium consisting of Eagle’s minimal essential medium (EMEM) and 199 medium (Chumakov Federal Scientific Center for Research and Development of Immunobiological Drugs of the Russian Academy of Sciences (Poliomyelitis Institute), Moscow, Russia) in a 1:1 ratio.

To obtain a compound **1**-resistant population of HSV-1/L_2_, compound **1** was introduced into the maintenance medium during serial passaging of the virus. The multiplicity of infection in passage 1 was 1 PFU/cell; the concentration of compound **1** was 10 µg/mL (~5 IC_50_). In subsequent passages, the multiplicity of infection was reduced to 0.1 PFU/cell, and the concentration of compound **1** was reduced to 4 µg/mL (~2 IC_50_). The sensitivity of the material of each passage was controlled using the virus-induced cytopathic effect (CPE) inhibition assay (see below). The concentration of compound **1** was gradually increased during passaging as the sensitivity of the virus decreased.

### 3.3. Cells

The Vero E6 (African green monkey (*Chlorocebus sabaeus*) kidney) cell culture was maintained by passaging in EMEM supplemented with 5% (*v*/*v*) fetal bovine serum (FBS) containing Hank’s salts, 2 mM L-glutamine, and 100 U/mL benzylpenicillin (PanEco, Moscow, Russia).

### 3.4. Antiviral Tests

The antiviral activities of compound **1** and anti-herpesvirus drugs were evaluated in vitro using the virus-induced CPE inhibition assay proposed by De Clerck, E. et al. [93], as we described in detail previously [60,94]. Serial dilutions of the corresponding drug with a multiplicity of two were prepared in 96-well Linbro plates (Flow Laboratories, Lancashire, UK) with the formed monolayer of the Vero E6 cell culture, then an equal volume of the virus-containing material (multiplicity of infection 0.1 PFU/cell) was added. A cell culture infected with the same multiplicity, but passaged without antiviral compounds in the maintenance medium, was used as a control. After 48 h, when 100% CPE developed in the control infected cultures, the IC_50_ and IC_95_ values were determined, providing 50% and complete inhibition of the development of viral CPE, respectively.

### 3.5. Cloning

To isolate virus clones resistant to compound **1**, monolayer cell cultures grown in culture flasks (*S* = 25 cm^2^; Corning, Glendale, AZ, USA) were infected with HSV-1/L_2_/R**_1_** passaged in the presence of compound **1** with a multiplicity of 0.01 PFU/cell. After adsorption for 1 h at 37 °C, cells were washed twice with sterile saline, then a 1:1 mixture of EMEM and 199 medium supplemented with 5% FBS containing Hank’s salts, 2 mM L-glutamine, and 0.4% agarose (Sigma-Aldrich, St. Louis, MO, USA) were added [59]. Cell cultures were incubated at 37 °C. After 48 h, material from individual (non-fused) plaques was collected using sterile microbiological loops (Biologix, Jinan, China), and the material obtained was used for re-infection of cell cultures. After three cycles of cloning, virus suspensions were obtained, which were genetically homogeneous material. The sensitivity of each clone to compound **1** was determined by virus-induced CPE inhibition assay. The resulting cloned material was stored at −80 °C.

### 3.6. Isolation of DNA from Herpesvirus-Infected Cells

DNA from virus-infected cells was isolated using the ExtractDNA Blood kit (Evrogen, Moscow, Russia). The concentration of isolated DNA was determined using a NanoDrop ND1000 spectrophotometer (Thermo Fisher Scientific, Waltham, MA, USA).

### 3.7. High-Throughput Sequencing of Herpesvirus Genomes

Preparation of libraries, high-throughput sequencing, and initial bioinformatics analysis were performed by Genoanalitika (Moscow, Russia). Isolated DNA samples were randomly fragmented by ultrasound treatment using a Covaris S2 US irradiator (Covaris, Woburn, MA, USA) to produce fragments with an average length of 200–250 base pairs. Fragmented DNA was used to prepare DNA libraries using the NEBNext Ultra II DNA Library Prep kit (New England Biolabs, Ipswich, MA, USA) according to the manufacturer’s instructions. Concentrations of the libraries were determined using a Qubit Fluorometer (ThermoFisher Scientific, Waltham, MA, USA) and the Qubit dsDNA HS Assay Kit (ThermoFisher Scientific, Waltham, MA, USA). The distribution of DNA fragment lengths in libraries was analyzed using the 2100 Bioanalyzer system (Agilent Technologies, Santa Clara, CA, USA) and Agilent High Sensitivity DNA Kit.

The prepared libraries were sequenced using the HiSeq 1500 System (Illumina, San Diego, CA, USA) using Illumina V4 PE chemistry, with one-end reads 70 base-long. Reads were mapped on NCBI Reference Sequence Human herpesvirus 1 strain 17, complete genome, accession no. NC_001806.2 (note that the current accession no. for the reference genome is JN555585.1).

### 3.8. Calculation Technique

All theoretical calculations were performed using the Schrodinger Small Molecule Drug Discovery Release 2021–4 software [95]. The main calculation strategy is to assess the affinity of compound **1** to potential binding sites.

#### 3.8.1. Preparation of Protein and Ligand

The geometrical parameters of the UL15–UL28–UL33 heterotrimer corresponding to the PDB code 6M5V [82] were downloaded from the non-commercial Protein Data Bank [96]. The model structure was prepared using the Protein prepwizard plugin [95] as follows: hydrogen atoms were added and minimized, side chains of AARs were edited, bond multiplicities were restored, and water molecules and other het-groups (and metal cations) were removed. The geometric parameters of the protein and the considered ligand were optimized using the OPLS4 force field method [97]. Protonation states were calculated during the protein preparation procedure with the use of the PROPKA method at pH 7.4.

#### 3.8.2. Binding Site Analysis

The ATPase domain, which includes the Walker loop AARs, as well as an alternative variant of ligand binding located closer to the central channel, was considered as a possible binding site.

#### 3.8.3. Molecular Docking

Molecular docking of the studied ligand was carried out using the protocol of forced docking (flexible docking) under the following conditions: flexible protein and ligand, grid-matrix size of 15 Å, and AARs within a radius of 5 Å from the ligand were optimized taking into account the influence of the ligand. The grid matrix was constructed either based on the position of the ADP reference ligand or on the basis of the position of key AARs located in the central channel of the domain or near it. All possible ligand conformations were taken into account during the molecular docking procedure, and the planarity of conjugated π-groups was enhanced. Extra-precision Glide docking was used. To take into account the possible movement of the side chains and improve the docking results, Trim side chain option based on B-factor was used: receptor van der Waals scaling was 0.7, and ligand van der Waals scaling was 0.5. The maximum number of positions was 10. We proceeded with the re-docking procedure for native ligand ADP. The geometric parameters of the ligand obtained as a result of molecular docking reproduced its position, deciphered electron microscopy (PDB code 6M5V). The RMSD value is 0.526 Å (see the Supplementary Materials, Figure S1).

Docking results were ranked by evaluating the following parameters: docking score (the main indicator of successful docking); ligand efficiency (LE), taking into account the atom-by-atom distribution of the scoring function; and the E-model clustering parameter, which includes the scoring function value, the energy of unbound interactions, and the parameters of the energy spent on the formation of the stacking of a compound in the binding site. In addition, we also evaluated the IFD score parameter (kcal/mol), which can be considered as the ligand–protein complex energy consisting of the total energy of the protein, the scoring function value, and the energy of the Coulomb interaction.

Binding energies (Δ*G*_MM-GBSA_) for ligand–protein complexes were estimated using the variable-dielectric generalized Born model, which incorporates residue-dependent effects; water was used as a solvent.

#### 3.8.4. Molecular Dynamics

For molecular dynamics simulations, we used the geometric parameters of wild-type and mutant-type heterotrimers, as well as ligand–protein complexes. The size of the buffer zone from the protein was 15 Å for all systems. The solvent model in all simulations was TIP3P; environment, NPT. Multistage equilibration with heating and restrained minimization of solvent and solute was used. The following options were selected for MD calculations: RESPA integrator 2.0 fs, Nose–Hoover chain ensemble, and 9.0 Å cutoff for long-range electrostatics. The period of the recorded dynamics simulation was 100 ns at a temperature of 310 K (37 °C). The protocol for preparing the system for simulation included preliminary minimization and balancing of the system components.

## 4. Conclusions

In summary, the selection of HSV-1 clones resistant to the lead compound (compound **1**) was performed; high-throughput sequencing of resistant clones was carried out, and the resulting reads were compared with the reference parent strain HSV-1/L_2_. The sequencing result identified a significant T321I mutation in the UL15 gene, which encodes the large subunit of terminase. The combination of the results of molecular docking and molecular dynamics simulations makes it possible to consider the pUL15 subunit as a biological target of the compound under study. We hypothesize that compound **1** can bind in the protein cavity near the central channel and the Walker B motif. We also do not exclude the possibility of compound **1** binding in the ATP domain; however, molecular dynamics results indicate that ligand binding at the ATP binding site is not stable. In addition, it is most likely that the location of compound **1** at an alternative binding site may be the reason for the T321I mutation. The ligand can affect the conformation of the side chains of functional amino acid residues of the central channel and the mobility of the protein secondary structure as a whole. The proposed mechanism of the antiviral action of compound **1** is then probably associated with the effect on the process of DNA binding during translocation. Of course, the results of molecular modeling are exclusively predictive. To confirm the binding site, it is necessary to decipher the geometric parameters of the ligand–protein complex by experimental methods, for example, X-ray diffraction analysis. However, this is a very time-consuming method.

The results obtained suggest that compound **1**, by binding to pUL15, affects the transferase complex. The UL15 protein, which is directly involved in the processing and packaging of viral DNA, is one of the crucial components of the HSV terminase complex. The loss of its functional activity leads to disruption in the formation of mature virions, so it is a promising drug target.

The first- and second-line anti-herpesvirus drugs (ACV, PCV, their prodrugs, CDV, and FOS) affect the viral DNA polymerase. The identification of novel antiviral agents that act on other targets will expand the possibilities of combating HSV infections, including those resistant to baseline antiviral drugs. The results of this work allow us to consider compound **1** as an alternative to modified nucleosides for the treatment of HSV-1 infection. Further study of compound **1** and related compounds as anti-herpesvirus agents is of undoubted interest.

## Figures and Tables

**Table 1:** Sensitivity of progeny HSV-1/L_2_ and compound **1**-resistant clones to a series of the approved antiherpetic agents * including drugs of practical importance in the Vero E6 cells.

Virus	IC_50_ (µg/mL)								
	Compound 1	ACV	PCV	GCV	BVDU	IDU	CDV	Ara-A	FOS
HSV-1/L_2_	1.95	0.39	0.78	0.56	0.098	3.9	3.9	15.6	31.25
HSV-1/L_2_/R_1_	9.75	0.39	0.78	1.12	0.114 ± 0.08	3.9	3.9	15.6	31.25
Clone 1	20.0	0.39	0.78	0.78	0.122	3.9	3.9	15.6	31.25
Clone 2	20.0	0.39	0.78	0.78	0.106 ± 0.008	3.9	3.9	15.6	31.25
Clone 3	20.0	0.39	0.78	0.89 ± 0.11	0.098	3.9	3.9	15.6	31.25

* ACV, acyclovir; PCV, penciclovir; GCV, ganciclovir; BVDU, 5-(2-bromovinyl)-2′-deoxyuridine; IDU, 5-iodo-2′-deoxyuridine; CDV, cidofovir; Ara-A, 9-β-D-arabinofuranosyladenine; FOS, foscarnet sodium.

**Table 2:** Percentage of reads mapped on reference herpesvirus genome (GenBank accession no. BK012101).

Sample	Total Reads × 10^6^	Software Used for Mapping	
		bowtie2	bowtie2 (Local)
Clone 1	5.75	15.33%	15.54%
Clone 2	5.11	23.39%	23.69%
Clone 3	5.36	23.80%	24.13%
Wild type	6.41	20.41%	20.67%

**Table 3:** List of mutations potentially responsible for herpesvirus resistance to compound **1**.

Gene Array	Product	Position on Reference Genome	Mutation in Genome	Product
TRL3-UL3	UL2 (uracil-DNA glycosylase)	9940	G→A	R19H
UL13.5-UL15	UL15 (large subunit of terminase)	29,982	C→T	T321I
UL27.5-UL30	UL30 (DNA polymerase)	65,203	C→T	E799D
UL37-UL39.6	UL37 (tegument protein)	81,716	C→T	R790H
US5-US7	US7 (glycoprotein I)	140,281	G→A	A165T

## Data Availability

Not applicable.

## Supplementary Materials

The following supporting information can be downloaded at: https://www.mdpi.com/article/10.3390/molecules28217375/s1, Table S1: Molecular docking results; Figure S1: Molecular re-docking procedure; Figure S2: Protein–ligand root-mean-square deviation (RMSD); Figure S3: Protein root-mean-square fluctuations (RMSF); Figures S4–S7: Protein–ligand contacts.
