# Supplementary material for: Large Subunit of the Human Herpes Simplex Virus Terminase as a Promising Target in Design of Anti-Herpesvirus Agents
Source: Molecules. 2023 Oct 31;28(21):7375. doi: 10.3390/molecules28217375 (PMC10649544; doi:10.3390/molecules28217375)
Supplement: Supplementary file 1 [file molecules-28-07375-s001.zip › molecules-2636586-SI.pdf]

Supplementary Materials

Large Subunit of the Human Herpes Simplex Virus Terminase as a Promising Target in Design of Anti-Herpesvirus Agents

Victor P. Krasnov [1,*], Valeriya L. Andronova [2], Alexander V. Belyavsky [3], Sophia S. Borisevich [4], George A. Galegov [2], Oleg F. Kandarakov [3], Dmitry A. Gruzdev [1], Olga A. Vozdvizhenskaya [1], Galina L. Levit [1]

1. Postovsky Institute of Organic Synthesis, Russian Academy of Sciences (Ural Branch), Ekaterinburg 620108, Russia
2. Gamaleya National Research Center for Epidemiology and Microbiology, Ministry of Health of the Russian Federation, Moscow 123098, Russia
3. Engelhardt Institute of Molecular Biology, Russian Academy of Sciences, Moscow 119991, Russia
4. Ufa Institute of Chemistry, Russian Academy of Sciences, Ufa 450078, Russia

Table of Contents

Table S1. Molecular docking results S2

Figure S1. Molecular re-docking procedure S3

Figure S2. Protein–ligand root-mean-square deviation (RMSD) S4

Figure S3. Protein root-mean-square fluctuations (RMSF) S5

Figures S4–S7. Protein–ligand contacts S6

Molecular Docking

Table S1. Molecular docking results

Site	ID complex	Pose	Docking score	E-model	IFD score	$\Delta G_{MM\text{-}BSGA}$ (kcal/mol)	H-bond	Other interactions	Clash
ATP domain	**1**–ATP-D	5	−8.9 (Δ = 2.7)	−91.04	−2739.8 (Δ = 3.3)	−61.1	R260<br>R261<br>G263<br>K264<br>S385	W266 – π-π<br>H194 – π-π	None
Central channel	**1**–CC	9	−3.3 (Δ = 1.0)	-36.71	−2732.7 (Δ = 3.2)	−32.8	H316	H316 – π-π	H316<br>D315
Loop 339–350	**1**–loop1<br>**1**–loop2	5<br>6	−8.3<br>−7.7	−79.4<br>−65.0	−2737.8<br>−2741.4	−56.3<br>−58.2	G347<br>E367<br>H340<br>N341<br>E320	F350 - π-π<br>none	I345<br>T368<br>T333<br>T321<br>none

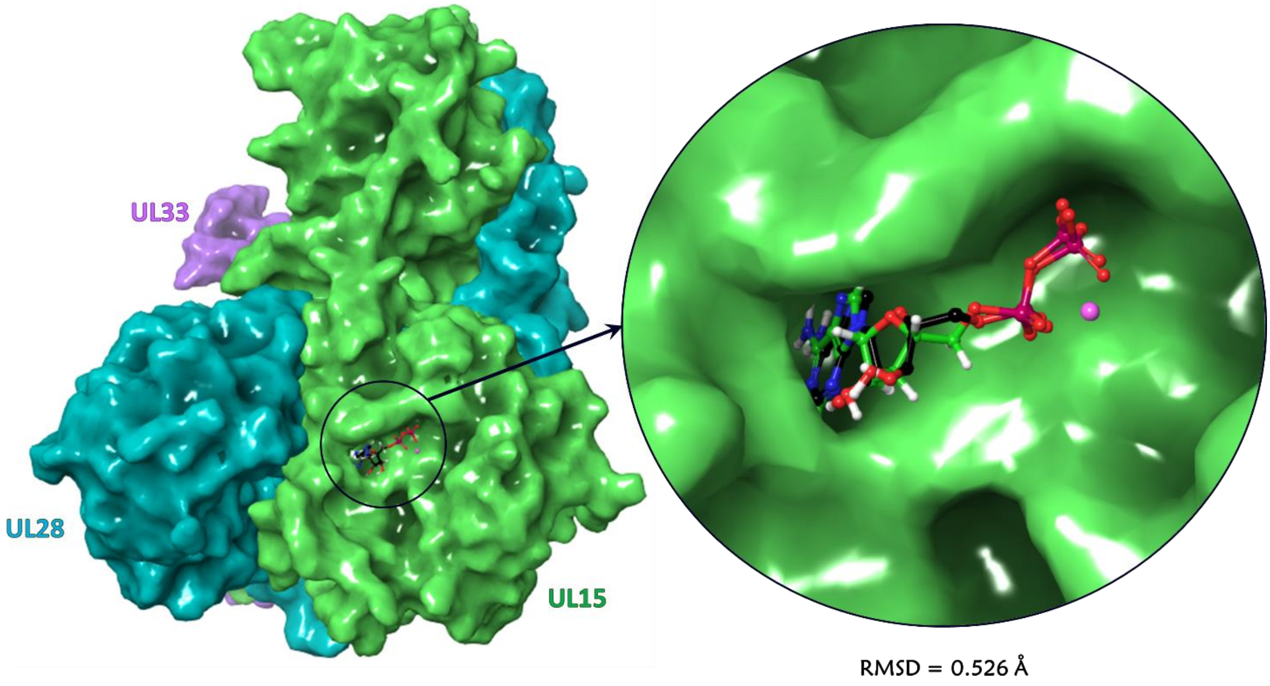

Figure S1. Molecular re-docking procedure. The green molecule corresponds to the geometric parameters obtained as a result of molecular docking. The black molecule corresponds to PDB code 6M5V.

Molecular Dynamics

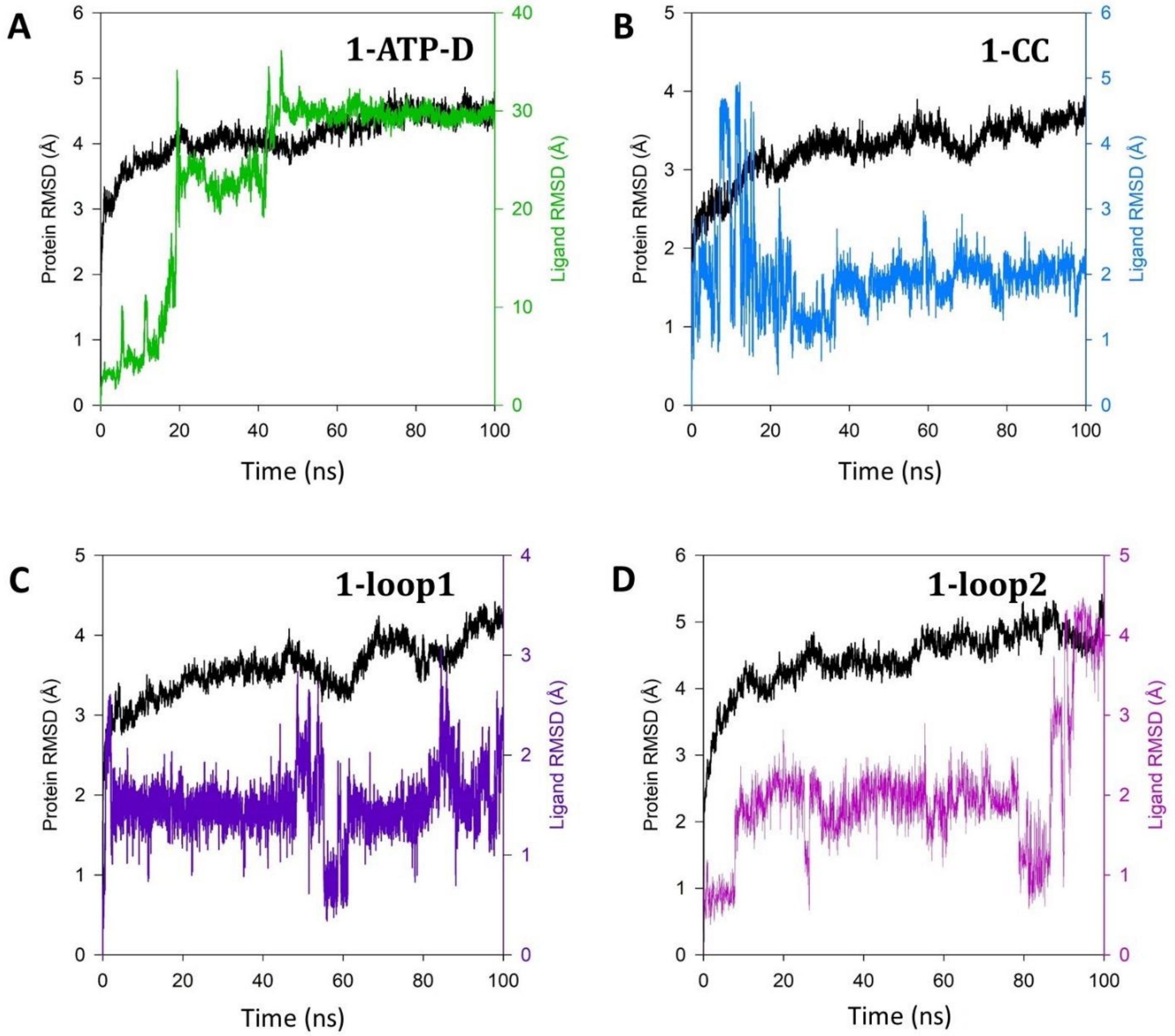

Figure S2. Protein–ligand root-mean-square deviation (RMSD): **A**, RMSD of the ligand and protein atoms in **1**–ATP-D complex; **B**, RMSD the ligand and protein atoms in **1**–CC complex; **C**, RMSD of the ligand and protein atoms in **1**–loop1 complex; **D**, RMSD of the ligand and protein atoms in **1**–loop2 complex.

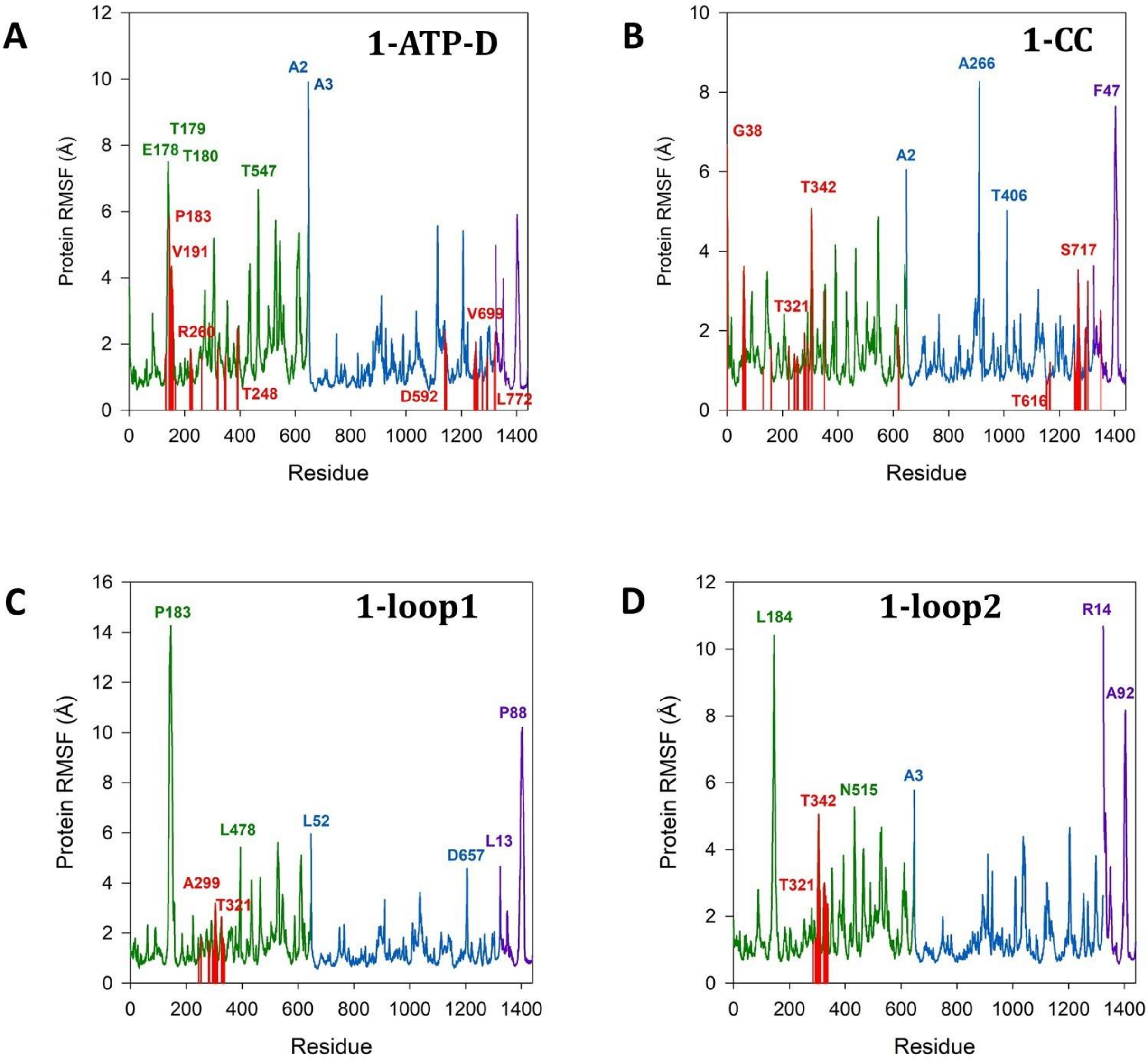

Figure S3. Protein root-mean-square fluctuations (RMSF): **A**, RMSF of the ligand and protein atoms in **1**–ATP-D complex; **B**, RMSF of the ligand and protein atoms in **1**–CC complex; **C**, RMSF of the ligand and protein atoms in **1**–loop1 complex; **D**, RMSF of the ligand and protein atoms in **1**–loop2 complex. Green plot corresponds to subunit UL15; blue, UL28; violet, UL33. Peaks indicate areas of the protein that fluctuate the most during the simulation. Protein residues that interact with the ligand are marked with red-colored vertical bars.

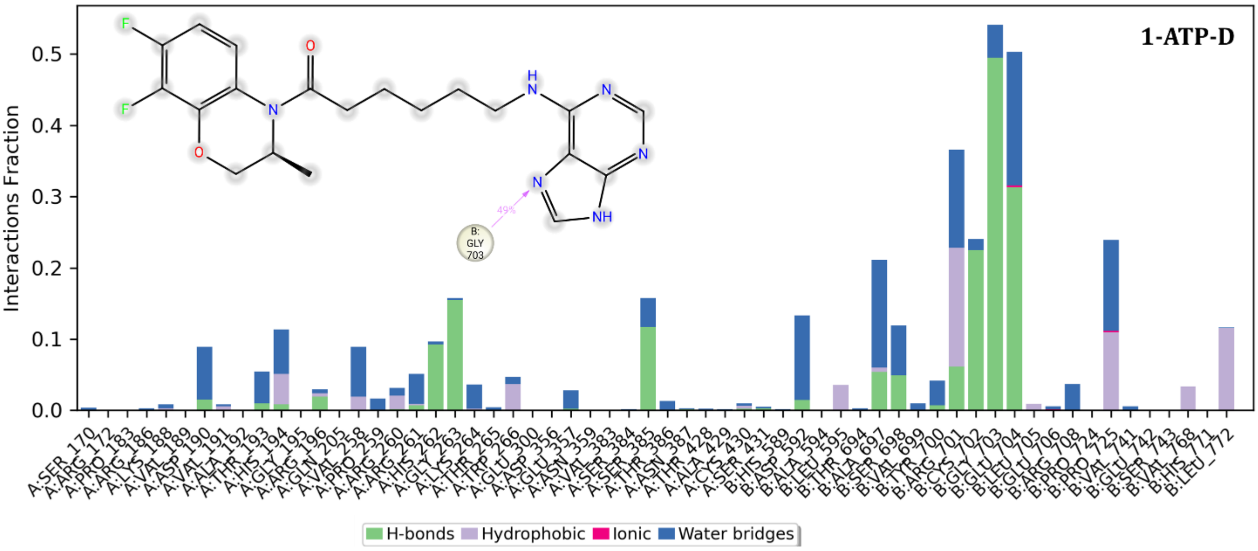

Figure S4. Protein–ligand contacts between atoms of ligand and protein in **1**–ATP-D complex.

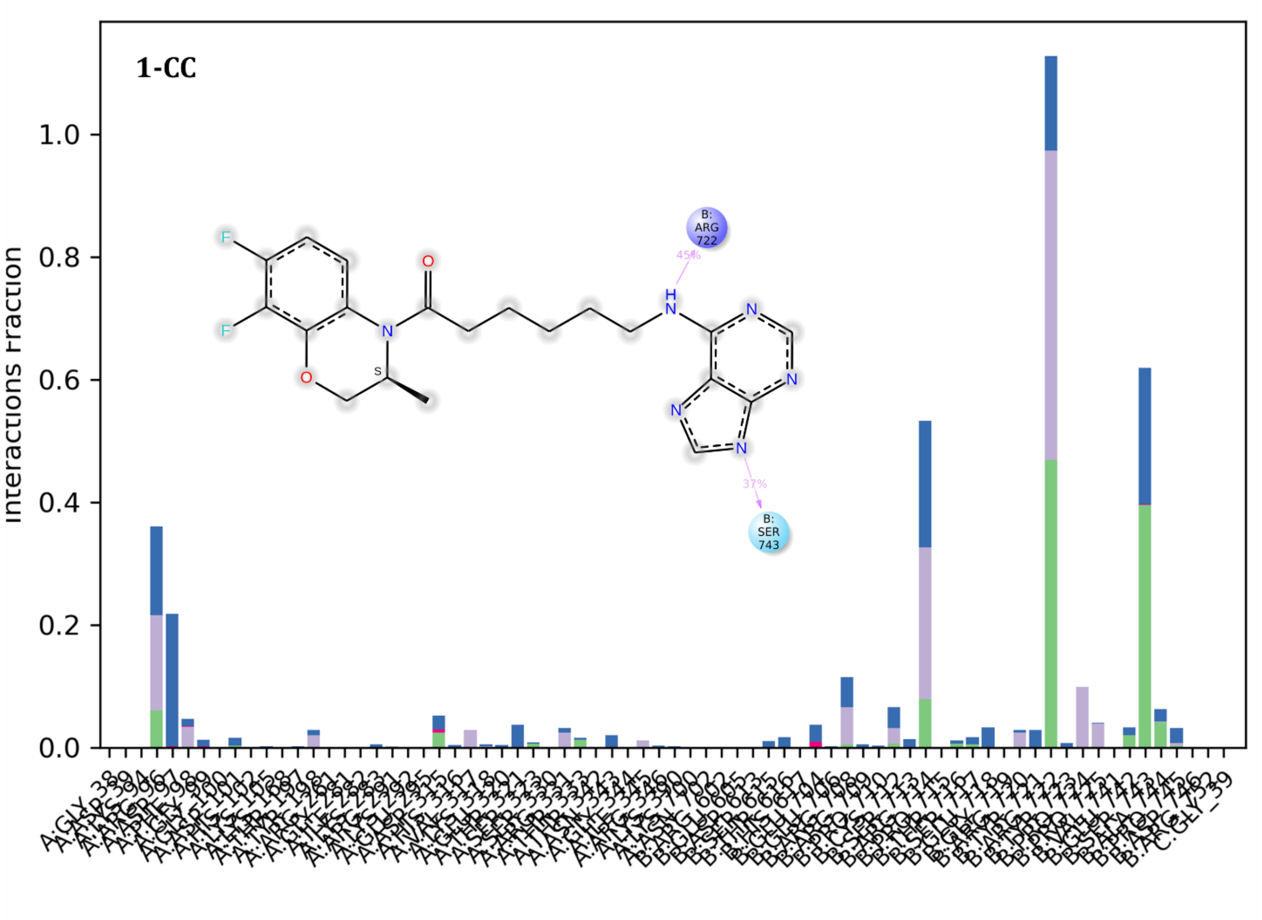

Figure S5. Protein–ligand contacts between atoms of ligand and protein in **1**–CC complex.

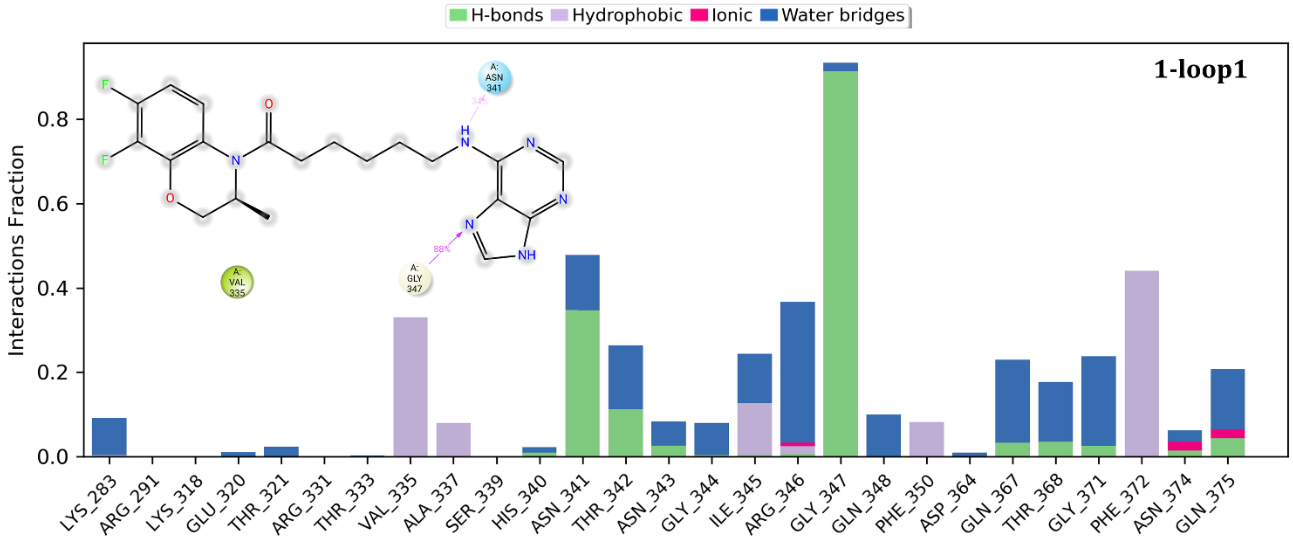

Figure S6. Protein–ligand contacts between atoms of ligand and protein in **1**–loop1 complex.

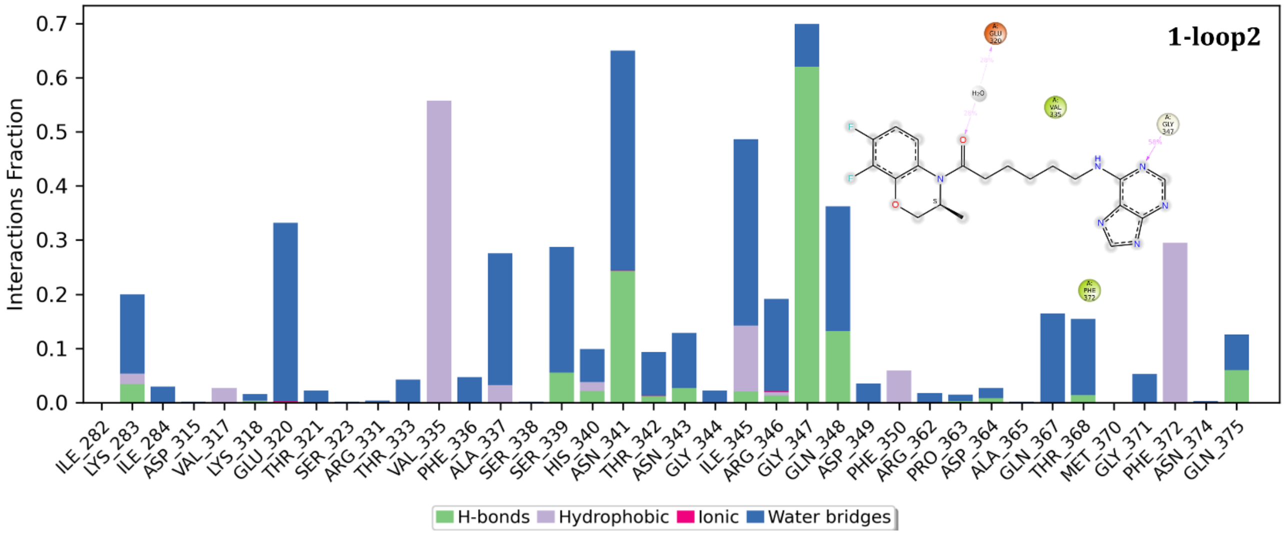

Figure S7. Protein–ligand contacts between atoms of ligand and protein in **1**–loop2 complex.
